# Meta-analysis of human gene expression in response to *Mycobacterium tuberculosis* infection reveals potential therapeutic targets

**DOI:** 10.1186/s12918-017-0524-z

**Published:** 2018-01-10

**Authors:** Zhang Wang, Seda Arat, Michal Magid-Slav, James R. Brown

**Affiliations:** 1 Computational Biology, Target Sciences, GlaxoSmithKline (GSK) R & D, Collegeville, PA 19426 USA; 2 Current address: The Jackson Laboratory, Farmington, CT 06032 USA

**Keywords:** Tuberculosis, Host-direct therapies, Gene expression signature, Parkinson’s disease, Drug repurposing

## Abstract

**Background:**

With the global emergence of multi-drug resistant strains of *Mycobacterium tuberculosis*, new strategies to treat tuberculosis are urgently needed such as therapeutics targeting potential human host factors.

**Results:**

Here we performed a statistical meta-analysis of human gene expression in response to both latent and active pulmonary tuberculosis infections from nine published datasets. We found 1655 genes that were significantly differentially expressed during active tuberculosis infection. In contrast, no gene was significant for latent tuberculosis. Pathway enrichment analysis identified 90 significant canonical human pathways, including several pathways more commonly related to non-infectious diseases such as the *LRRK2* pathway in Parkinson’s disease, and *PD-1*/*PD-L1* signaling pathway important for new immuno-oncology therapies. The analysis of human genome-wide association studies datasets revealed tuberculosis-associated genetic variants proximal to several genes in major histocompatibility complex for antigen presentation. We propose several new targets and drug-repurposing opportunities including intravenous immunoglobulin, ion-channel blockers and cancer immuno-therapeutics for development as combination therapeutics with anti-mycobacterial agents.

**Conclusions:**

Our meta-analysis provides novel insights into host genes and pathways important for tuberculosis and brings forth potential drug repurposing opportunities for host-directed therapies.

**Electronic supplementary material:**

The online version of this article (doi: 10.1186/s12918-017-0524-z) contains supplementary material, which is available to authorized users.

## Background

The causative agent of tuberculosis (TB), the bacterium *Mycobacterium tuberculosis* (*Mtb*), infects about one third of the world’s population. In 2012, an estimated 8.6 million individuals progressed to active disease and 1.3 million died [1]. The prolonged duration of *Mtb* infection as well as the alarming emergence of multi-drug resistant strains makes the development of new and effective anti-tubercular therapeutics a global health priority [2, 3].

The severity of TB is largely dependent upon the modulation of human cellular and immunity pathways by the *Mtb* pathogen. The majority of infected patients develop latent TB (LTB) infection, in which they are asymptomatic with no clinical evidence of disease for years or decades [4]. During latent infection, *Mtb* is phagocytosed by macrophages, which trigger host immune response involving the recruitment of additional macrophages and monocytes that ultimately form an organized structure called granuloma [5]. *Mtb* is dormant and non-replicative within granuloma which suppresses the immediate threat of active infection while evading further immune response [6]. Approximately 5–10% of LTB patients will go on to develop active pulmonary TB (PTB), in which *Mtb* returns to the replication mode and provokes an active host immune response. When the granuloma breaks down, sequestered *Mtb* can be released into the airway and becomes transmissible to other hosts.

Currently, the standard TB therapy involves a regimen of four antibiotics taken during an initiation phase of two months and a continuation phase of 4–7 months [7, 8]. Despite a high efficacy at the onset of administration, the confounded intervention and prolonged treatment period present challenges for patient compliance and potentially foster the emergence of multidrug-resistance of *Mtb*. Thus, it is imperative to develop more effective therapeutic approaches such as host-directed therapies. Targeting the host has its advantages in potentially being less susceptible to the drug resistance problem, with greater opportunities for repositioning known drugs to new indications.

Recent genome-wide studies of host-pathogen interactions provide new insights into human genes and pathways modulated by pathogen infection and proliferation. Multiple studies have generated extensive human microarray gene expression datasets for subjects infected by *Mtb* with the overall objective of developing diagnostic biomarkers [9–11]. For example, by comparing 14 public human gene expression datasets for LTB, PTB and other respiratory diseases, Sweeney et al. identified a three-gene set that was robustly diagnostic for PTB [12]. However, to our knowledge, no systematic search for potential host therapeutic targets against TB has been published.

Previously, we applied integrated analysis to human gene expression data in response to respiratory bacterial and viral infections in order to identify novel host targets and potential drug repurposing opportunities [13, 14]. In this study, we performed a statistical meta-analysis on human gene expression data available for *Mtb* infection to identify common human transcriptomic signatures characteristic of TB. The advantage of meta-analysis lies in its capability to alleviate potential study-specific biases and enhance statistical power to identify robust expression signature through increased sample size. We leveraged other evidence such as human genetic disease associations and drug-repurposing analyses to prioritize individual targets and compounds. We identified multiple host genes and pathways that were significantly altered in TB and propose several potential drug candidates for repurposing.

## Methods

### Data sources, filtering and selection

Human microarray gene expression datasets in response to *Mtb* infection were retrieved from the National Center for Biotechnology Information’s (NCBI) Gene Expression Omnibus (GEO) database (http://www.ncbi.nlm.nih.gov/geo/), by searching ‘Tuberculosis’, ‘*Homo sapiens*’ and ‘expression profiling by array’. GEO datasets were then filtered based on the following criteria: 1) the gene expression profile was exclusively derived from human cells of tuberculosis patients and probed using a human-based genome array platform; 2) there was at least one control group and patient group in the dataset, with the control group consisting of only healthy subjects, and the patient group consisting of patients only infected by *Mtb* without other diseases such as HIV; 3) each patient and control group had at least three samples.

Raw gene expression data, study design table and annotation table of each dataset were obtained from the GEO database and processed using ArrayStudio v8.0 (OmicSoft, USA). All datasets retrieved are microarray datasets except for GSE41055, which is an exon array and was excluded from further analysis. Several datasets (GSE42834, GSE56153, GSE31348 and GSE36238) were further excluded due to both a noisy kernel density plot and low within group pairwise correlation (correlation cutoff 0.9 as suggested in [15], Additional file 1), but were included as independent datasets for validation purposes. After quality filtering, nine microarray datasets (GSE19435, GSE19439, GSE19444, GSE28623, GSE29536, GSE34608, GSE54992, GSE62525 and GSE65517) from either whole blood or peripheral blood mononuclear cells (PBMCs) were retained for further analysis.

We also searched for available datasets of individual human immune cells under in vitro *Mtb* challenge. We identified three datasets of human dendritic cells (GSE34151, GSE360 and GSE53143) and four datasets of THP-1 human leukemia monocytic cells (GSE17477, GSE29628, GSE51029 and GSE57028), and processed them separately. By comparing gene expression profiles derived from PBMCs or whole blood to those of immune cells, we could evaluate how well blood gene expression patterns recapitulate those of immune cells during *Mtb* infection.

Quality Control (QC) analysis was performed for each of the nine datasets as described previously [13]. Briefly, samples that were outliers in at least two of the four assessments: 1) kernel density; 2) Principal Component Analysis (PCA); 3) Median Absolute Deviation (MAD) score and; 4) within group pairwise correlation, were excluded from downstream analyses. For example, samples GSM484458 and GSM484465 in GSE19439, and GSM851876 and GSM851889 in GSE34608 were excluded because they were outliers in both MAD score and pairwise correlation (Additional file 2). Samples GSM484369 from GSE19435, and GSM484609 and GSM484629 from GSE19444, were outliers in both pairwise correlation and kernel density. Samples irrelevant to our study design (such as samples from sarcoidosis patients in GSE34608) were also excluded from each dataset. In total, 106 control, 76 LTB and 131 PTB samples from the nine datasets passed the QC criteria for statistical meta-analysis (Table 1).

**Table 1:** List of GEO datasets in the meta-analysis

Dataset	Cell type	PMID	Platform	Samples				DEGs	
				PTB	LTB	Control	Outlier	PTB	LTB
GSE19435	Whole blood	20725040	Illumina	12	0	7	1	3881	NA
GSE19439	Whole blood	20725040	Illumina	17	13	6	2	461	0
GSE19444	Whole blood	20725040	Illumina	20	20	12	0	1916	0
GSE28623	Whole blood	22046420	Agilent	41	23	35	9	3974	0
GSE29536	Whole blood	24069364	Illumina	9	0	6	0	2719	NA
GSE34608	Whole blood	22547807	Agilent	8	0	18	2	9694	NA
GSE54992	PBMC	24647646	Affymetrix	9	6	6	3	3741	0
GSE62525	PBMC	26818387	Phalanx	12	14	13	3	8719	6934
GSE65517	PBMC	25992611	Illumina	3	0	3	0	414	NA
GSE34151	Dendritic	22233810	Illumina	129	0	126	4	5716	NA
GSE360	Dendritic	12663451	Affymetrix	2	0	2	0	479	NA
GSE53143	Dendritic	24482540	Illumina	8	0	10	0	3461	NA
GSE17477	THP-1	NA	Affymetrix	4	0	4	0	251	NA
GSE29628	THP-1	22675550	Affymetrix	5	0	1	0	174	NA
GSE51029	THP-1	NA	Agilent	123	0	69	14	3858	NA
GSE57028	THP-1	24899504	Affymetrix	3	0	3	0	2431	NA

List of patient blood and in vitro dendritic and THP-1 datasets in this study, and the number of samples and DEGs in each dataset

### Data processing and statistical meta-analysis

Data processing and statistical meta-analysis were performed using the web-based tool NetworkAnalyst [16]. Briefly, probe identifiers (IDs) from different microarray platform were converted to Entrez gene IDs. If more than one probe mapped to a gene, the average expression value of these probes was used for that gene. The gene expression level in each comparison group was log2 transformed and auto-scaled. Differential expression analysis for individual datasets was performed using the limma approach [17], with a false discovery rate (FDR)-adjusted *P*-value cutoff of 0.05.

The study-specific batch effects were adjusted for the pre-processed datasets using the ComBat procedures as described in [18, 19]. Batch effects corrected individual datasets were then combined and a statistical meta-analysis was performed using INMEX within NetworkAnalyst [16]. We chose to use the combined effect size method for the meta-analysis which generates more conservative and biologically consistent results than the alternative *P*-value combination method [18, 20]. The random effect model that incorporates cross-study heterogeneity was selected for the meta-analysis because of significant heterogeneity detected using Cochrans’ Q test [21]. Differentially expressed genes (DEGs) were generated using an FDR-adjusted *P*-value cutoff 0.01 in the meta-analysis. A subset of DEGs with absolute combined effect size greater than 1.5 was selected for genetic variant and DrugBank analyses.

### Validation of meta-analysis

To evaluate the robustness of the meta-analysis results, we validated the DEGs in four independent datasets (GSE42834, GSE56153, GSE31348 and GSE36238) using PCA and Partial Least Square Discriminant Analysis (PLS-DA). PLS-DA is a multivariate analysis that establishes a linear regression model between the observed categorical variable and multiple predicting variables. Expression data were scaled to unit variance. Collinearity was addressed using pairwise Pearson’s correlation test. One gene was selected as representative for each group of genes whose expression values were strongly mutually correlated (R^2^ > 0.5). Significant model components were selected by 7-fold cross validation. The model performance was evaluated in terms of R^2^, Q^2^ and the area under the Receiver Operating Characteristic (ROC) curve (AUC). R^2^ measures the percent of variation of the categorical variable explained by the model. Q^2^ measures the percent of variation of the categorical variable predicted by the model through 7-fold cross-validation. The ROC curve plots the true positive rate (sensitivity) against the false positive rate (1-specificity) when varied threshold is applied. The AUC values were calculated and compared using pROC package in R [22].

To explore possible prognostic value of the DEGs, a Kaplan-Meier survival analysis was performed using the top 50 DEGs in the meta-analysis on the largest lung cancer cohort (Lung Meta-base: 1053 samples, 6 cohorts, 22 K genes) in SurvExpress [23], as significant comorbidities have been observed between lung cancer and TB [24].

### Pathway enrichment analysis

Pathway enrichment analysis was performed for all DEGs from the meta-analysis using MetaCore/MetaBase (GeneGo) v5.0 (Thomson Reuters, https://portal.genego.com/). The *P*-value for each of the 1480 human canonical pathways in MetaCore was generated using a hypergeometric test with an FDR *P*-value cutoff of 0.01 [25]. Protein-protein interaction (PPI) network analysis was performed based on the STRING interactome using NetworkAnalyst [16, 26]. Experimental evidence was required with a default confidence score 0.9 for any interaction between two genes. The minimum network mode was chosen for display purposes.

### Genetic variant analysis

We searched for any TB-associated single nucleotide polymorphisms (SNPs) proximal to the DEGs. Genetic variants significantly associated with TB (genome-wide significant *P*-value < 5e-8) were obtained from the Genome-Wide Repository of Associations Between SNPs and Phenotypes (GRASP, http://grasp.nhlbi.nih.gov/) [27]. As usually few non-synonymous coding variants were associated with the genes, we also searched for regulatory SNPs for the DEGs by obtaining their enhancer regions from FANTOM5, a comprehensive resource on active transcripts, transcription factors, enhancers and promoters in mammalian primary cell types and cancer cell lines [28]. The genomic region in linkage disequilibrium (LD) with each SNP was obtained from SNAP (https://www.broadinstitute.org/mpg/snap/) [29]. Any overlap between the genomic locations of a DEG or its FANTOM5 enhancers and the LD region of a SNP would indicate a potential association between the corresponding gene and the SNP.

### DrugBank and connectivity map analysis

Drug-target links between public compounds and the DEGs were obtained from DrugBank Version 4.5 (http://www.drugbank.ca/) [30]. Only launched drugs with experimental or clinical evidence of direct interaction with the encoded gene product were included. Drug-target links were excluded if the drugs have unknown pharmacological actions or pharmacological actions of the same direction to the differential expression of the targets in TB signature.

Drug repurposing analysis was performed using the Connectivity Map (CMAP, https://www.broadinstitute.org/cmap/) [31]. The 250 top-ranked and 250 bottom-ranked DEGs from the meta-analysis were used for generating gene expression signature as recommended by Iorio et al. [32], which was then compared against the gene expression signatures of all Broad CMAP compounds. Significant CMAP compounds against TB were identified based on the following criteria: 1) The enrichment score of the compound was less than 0 for inversely matched drug and disease signatures; 2) FDR-adjusted *P*-value < 0.05; 3) The compound specificity was less than 0.1. The target, mechanism of action, therapy area and indication for each compound were obtained from PubChem (http://pubchem.ncbi.nlm.nih.gov/) and DrugBank [30]. Antibacterial compounds and compounds with no clinical use were excluded from the drug annotation.

## Results

### Statistical meta-analysis for differentially expressed genes during PTB

**Fig. 1:**
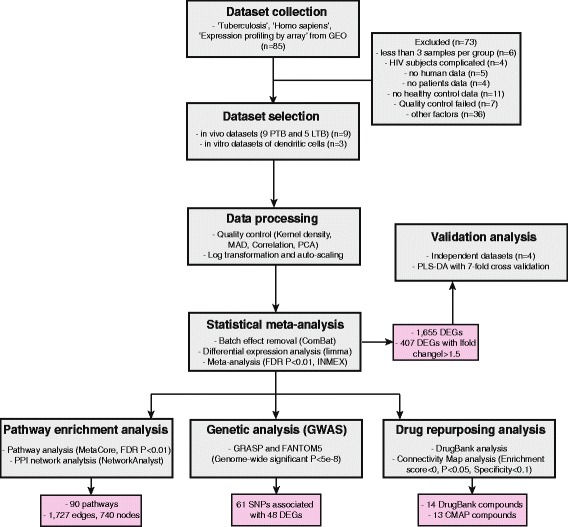
Flowchart of the statistical meta-analysis of human gene expression in response to *Mtb* infection. The process consists of eight major steps which were detailed in the grey boxes. The output of each data analysis step was indicated in the corresponding pink box. Detailed criteria for each major step were described in Methods

We employed an iterative process of database querying, filtering and computational analyses for all datasets (Fig. 1). At the time of this study (October 2016), there were 85 GEO microarray datasets available for TB-related host responses (Additional file 3). After filtering based on dataset inclusion criteria (see Methods), a final set of nine in vivo patient datasets including nine PTB and five LTB comparisons was selected for downstream analyses (Table 1). We also identified three datasets of human dendritic cells and four datasets of human THP-1 cells under in vitro *Mtb* challenge, and compared their expression signatures to the TB patient signatures. The complete list of the 85 GEO datasets and their annotations are shown in Additional file 3.

**Fig. 2:**
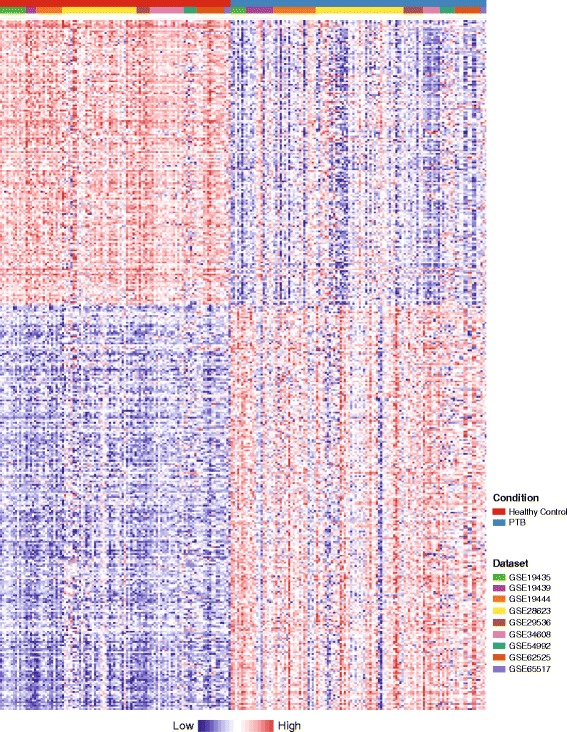
The heatmap of the subset 407 DEGs identified in the meta-analysis. For each DEG, its normalized expression value in each sample of the nine datasets was indicated in the heatmap. Two hundred forty one genes were up-regulated and 166 genes were down-regulated. The genes were clustered using the Ward’s method [56]. The samples were grouped first by comparison group then by individual studies

Statistical meta-analysis of the nine datasets was performed using NetworkAnalyst [16]. For PTB comparisons, a total number of 16,703 genes common to all nine datasets were identified and a combined master dataset was generated. Batch effect was corrected using the ComBat approach [19]. PCA indicates that all samples were tightly clustered according to the specific study prior to the batch effect correction. After the correction, samples from different studies were intermixed and now clustered primarily based on PTB and control groups (Additional file 4). The samples do not cluster based on sample type in either PCA plot before or after batch effect correction (Additional file 4). A total of 1655 DEGs were identified in the meta-analysis under an FDR-adjusted *P*-value cutoff 0.01 (Additional file 5). Of these, a subset of 407 DEGs had an absolute combined effect size as a reference for the log2 fold change greater than 1.5 (Fig. 2). For LTB comparisons, no gene was retained using the same criterion in the meta-analysis of the five datasets.

### Identification of significantly enriched pathways

**Fig. 3:**
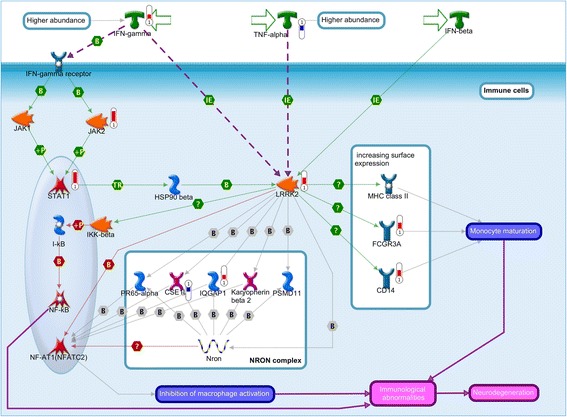
Pathway map for “*LRRK2* and immune function in Parkinson’s disease”. Significant up-regulation of genes was denoted as up-pointing bars colored in red, and significant down-regulation of genes was denoted as down-pointing bars colored in blue. The length of the colored bar was proportional to the fold change of the gene in the meta-analysis

**Fig. 4:**
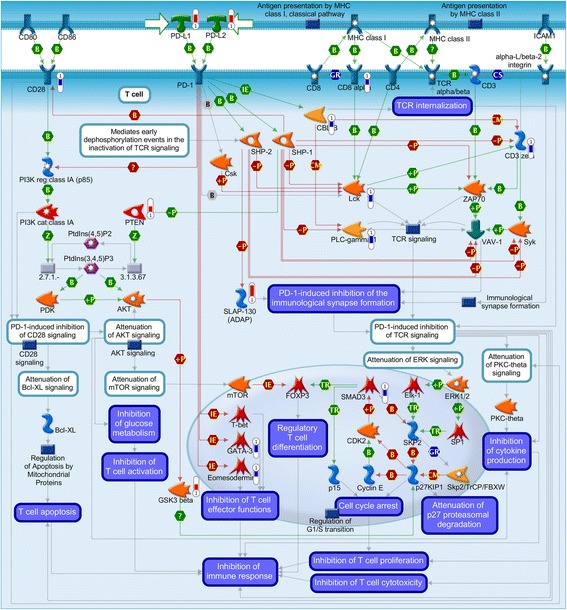
Pathway map for “Inhibitory *PD-1* signaling in T cells”. Significant up-regulation of genes was denoted as up-pointing bars colored in red, and significant down-regulation of genes was denoted as down-pointing bars colored in blue. The length of the colored bar was proportional to the fold change of the gene in the meta-analysis

The 1655 significant DEGs were then analyzed for enriched functional pathways using MetaCore v5.0, a high quality, manually curated protein-interaction database supported by ‘small experiment’ evidence. In total, 90 human canonical pathways were significantly enriched from the DEGs (FDR-adjusted *P* < 0.01) (Table 2), many of which were involved in host immune response. The most significant pathway was ‘*IFN* type I signaling pathway’ (FDR-adjusted *P* = 3e-9, Additional file 6) which is a known host response to TB [33], with 17 DEGs in 37 pathway components. Notably, there was significant enrichment for several pathways more commonly associated with non-infectious diseases. These include the leucine-rich repeat kinase 2 (*LRRK2*) pathway in Parkinson’s disease (FDR-adjusted *P* = 9.4e-5, Fig. 3) and *PD-1*/*PD-L1* (Programmed Death 1/PD-Ligand 1) signaling pathway (FDR-adjusted *P* = 6.5e-5, Fig. 4). A few pathways were associated with other diseases such as asthma, chronic obstructive pulmonary disease and dermatitis, albeit all with sparsely connected genes.

**Table 2:** List of 90 significantly enriched human pathways in the meta-analysis

Map	Major Process	- log (*P*)	DEGs	In dendritic data	In THP-1 data
Attenuation of IFN type I signaling in melanoma cells	Cancer	8.53	17	Y	N
Bacterial infections in CF airways	CF pathways	7.66	18	N	Y
Role of PKR in stress-induced antiviral cell response	Immune response	6.41	18	Y	Y
B cell signaling in hematological malignancies	Immune response	6.29	21	N	N
Bacterial infections in normal airways	Immune response	5.93	16	N	N
TLR2 and TLR4 signaling pathways	Immune response	5.82	17	N	Y
Role of CD8+ Tc1 cells in COPD	COPD	4.95	14	Y	Y
SLE genetic marker-specific pathways in B cells	Immune response	4.74	21	N	Y
Release of pro-inflammatory mediators and elastolytic enzymes by alveolar macrophages in COPD	COPD	4.7	11	N	Y
G-CSF-induced myeloid differentiation	Development	4.43	11	N	Y
Inter-cellular relations in COPD (general schema)	COPD	4.43	11	Y	Y
IL-1 signaling pathway	Immune response	4.28	13	N	Y
Inflammatory mechanisms of pancreatic cancerogenesis	Cancer	4.25	16	Y	Y
Antiviral actions of Interferons	Immune response	4.25	14	N	N
Role of fibroblasts and keratinocytes in the elicitation phase of allergic contact dermatitis	Dermatitis	4.25	10	Y	Y
Inhibitory PD-1 signaling in T cells	Immune response	4.19	14	Y	N
Role of iNKT and B cells in T cell recruitment in allergic contact dermatitis	Dermatitis	4.07	12	N	N
LRRK2 and immune function in Parkinson’s disease	Parkinson	4.03	9	N	N
TLR5, TLR7, TLR8 and TLR9 signaling pathways	Immune response	4.03	13	N	Y
Chemokines in inflammation in adipose tissue and liver in obesity, type 2 diabetes and metabolic syndrome X	Immune response	4.03	13	N	Y
Neutrophil-derived granule proteins and cytokines in asthma	Asthma	3.94	13	N	N
Cigarette smoke-mediated attenuation of antibacterial and antivirus immune response	Immune response	3.92	10	Y	Y
Prostate Cancer: candidate susceptibility genes in inflammatory pathways	Cancer	3.92	10	N	N
Inhibition of apoptosis in multiple myeloma	Cancer	3.86	11	Y	N
SLE genetic marker-specific pathways in antigen-presenting cells (APC)	Immune response	3.79	17	Y	Y
Proinflammatory cytokine production by Th17 cells in asthma	Asthma	3.61	13	N	N
Antigen presentation by MHC class I, classical pathway	Immune response	3.53	13	Y	N
NK cells in allergic contact dermatitis	Dermatitis	3.43	10	N	Y
Inflammatory response in ischemia-reperfusion injury during myocardial infarction	Stem cells	3.36	8	N	Y
Putative pathways of activation of classical complement system in major depressive disorder	Complement activation	3.25	9	N	N
Th1 and Th17 cells in an autoimmune mechanism of emphysema formation in smokers	Signal transduction	3.25	9	N	Y
NF-kB activation pathways	Signal transduction	3.14	12	N	Y
iNKT cell-keratinocyte interactions in allergic contact dermatitis	Dermatitis	3.13	10	N	N
Apo-2 L(TNFSF10)-induced apoptosis in melanoma	Cancer	2.98	11	Y	Y
IFN alpha/beta signaling pathway	Immune response	2.95	8	Y	N
EGFR signaling pathway	Development	2.95	14	N	Y
Th17 cells in CF	CF pathways	2.95	12	N	N
ERBB family and HGF signaling in gastric cancer	Cancer	2.95	12	N	Y
Interleukins-induced inflammatory signaling in normal and asthmatic airway epithelium	Immune response	2.95	9	N	N
Role of keratinocytes and Langerhans cells in skin sensitization	Skin sensitization	2.87	8	N	N
Transcription regulation of granulocyte development	Development	2.87	9	N	Y
The role of KEAP1/NRF2 pathway in skin sensitization	Skin sensitization	2.87	9	N	Y
Activation of ACTH production in pituitary gland in major depressive disorder	Signal transduction	2.87	9	N	N
IFN gamma signaling pathway	Immune response	2.84	12	N	Y
Memory CD8+ T cells in allergic contact dermatitis	Dermatitis	2.84	10	N	Y
MIF in innate immunity response	Immune response	2.84	10	N	N
The innate immune response to contact allergens	Immune response	2.81	9	N	N
IL-5 signaling via JAK/STAT	Immune response	2.81	12	Y	Y
Cytokine-mediated regulation of megakaryopoiesis	Development	2.81	12	Y	N
Inflammasome in inflammatory response	Immune response	2.72	9	N	N
TLR ligands	Immune response	2.72	9	N	N
Rheumatoid arthritis (general schema)	Others	2.71	11	Y	N
Apoptotic TNF-family pathways	Apoptosis and survival	2.7	10	Y	N
Neutrophil resistance to apoptosis in COPD and proresolving impact of lipid mediators	COPD	2.64	12	Y	Y
Regulation of proinflammatory cytokine production by Th2 cells in asthma	Asthma	2.64	10	N	N
Role of cell adhesion in vaso-occlusion in Sickle cell disease	Sickle cell disease	2.64	10	N	N
Release of pro-inflammatory factors and proteases by alveolar macrophages in asthma	Asthma	2.64	10	Y	Y
HMGB1/TLR signaling pathway	Immune response	2.57	9	N	Y
Role of TLR signaling in skin sensitization	Skin sensitization	2.57	10	N	Y
Inhibition of neutrophil migration by proresolving lipid mediators in COPD	COPD	2.54	13	N	N
Role of PKR in stress-induced apoptosis	Apoptosis and survival	2.54	11	Y	N
TLRs-mediated IFN-alpha production by plasmacytoid dendritic cells in SLE	SLE	2.54	11	N	Y
Role of IFN-beta in activation of T cell apoptosis in multiple sclerosis	Multiple sclerosis	2.54	8	Y	N
HSP60 and HSP70/ TLR signaling pathway	Immune response	2.48	11	N	Y
T cell receptor signaling pathway	Immune response	2.41	11	N	N
Integrin inside-out signaling in T cells	Cell adhesion	2.39	13	Y	N
HGF signaling pathway	Development	2.37	10	Y	N
Prolactin/ JAK2 signaling in breast cancer	Cancer	2.32	7	N	Y
IFN-gamma and Th2 cytokines-induced inflammatory signaling in normal and asthmatic airway epithelium	Asthma	2.26	9	N	Y
TNF-alpha and IL-1 beta-mediated regulation of contraction and secretion of inflammatory factors in normal and asthmatic airway smooth muscle	Asthma	2.26	12	N	Y
Antigen presentation by MHC class II	Immune response	2.26	5	N	N
Integrin inside-out signaling in neutrophils	Cell adhesion	2.26	13	N	N
Role of B cells in SLE	SLE	2.26	11	Y	N
Th17 cells in CF (mouse model)	CF pathways	2.26	10	N	N
LPS-induced platelet activation	Immune response	2.24	7	N	N
IL-18 signaling	Immune response	2.15	11	N	Y
Inhibition of apoptosis in gastric cancer	Cancer	2.15	9	Y	Y
CD8+ Tc1 cells in allergic contact dermatitis	Dermatitis	2.15	7	Y	N
Role of IL-8 in colorectal cancer	Cancer	2.15	6	N	N
Schema: Initiation of T cell recruitment in allergic contact dermatitis	Dermatitis	2.15	6	N	N
Inhibition of WNT5A-dependent non-canonical pathway in colorectal cancer	Cancer	2.15	6	N	Y
Function of MEF2 in T lymphocytes	Immune response	2.15	10	N	Y
Caspase cascade	Apoptosis and survival	2.15	8	Y	Y
Regulation of Tissue factor signaling in cancer	Cancer	2.1	9	N	N
Regulatory T cells in murine model of contact hypersensitivity	Others	2.07	7	N	N
Production and activation of TGF-beta in airway smooth muscle cells	Signal transduction	2.07	8	N	N
Development_Growth hormone signaling via STATs and PLC/IP3	Development	2.07	8	N	Y
Apoptotic pathways and resistance to apoptosis in lung cancer cells	Cancer	2.04	10	Y	Y
Cigarette smoke-induced inflammatory signaling in airway epithelial cells	Signal transduction	2	8	N	Y
IL-12-induced IFN-gamma production	Immune response	2	8	N	N

List of 90 significantly enriched human pathways in the meta-analysis, their major processes, −log (*P*-value), number of DEGs in the pathways, and whether they were identified in in vitro dendritic or THP-1 datasets

**Fig. 5:**
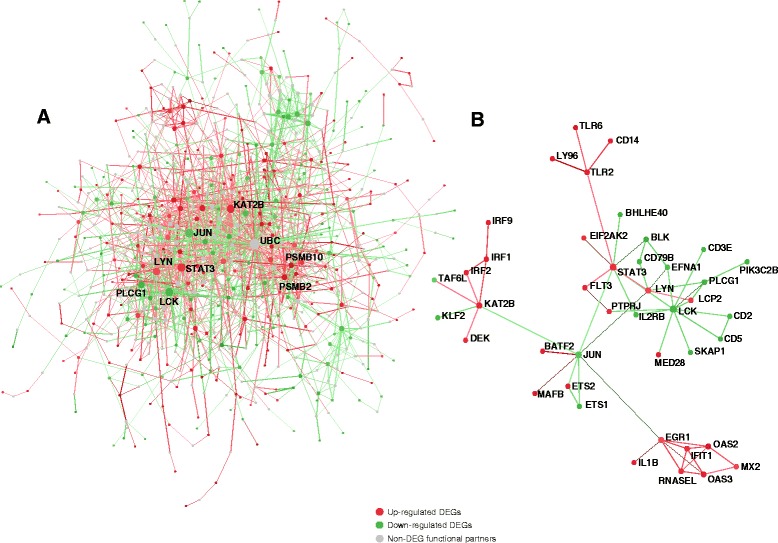
Protein-protein interaction network of the 1655 DEGs in the meta-analysis. **a** The subnetwork of all DEGs including their functional partners. Each node represents a gene and each edge represents an interaction between two genes supported by experimental evidence. The up-regulated genes were colored in red. The down-regulated genes were colored in green. The non-DEG functional partners were colored in grey. The minimum network mode was chosen for display purposes. **b** The subnetwork of only DEGs exclusive of their functional partners

To explore the interactive relationships between the 1655 DEGs, we performed a protein-protein interaction network analysis based on the STRING interactome database using NetworkAnalyst [16, 26]. We obtained a subnetwork containing 1727 edges and 740 nodes, 364 of which were DEGs (Fig. 5a). Among the DEGs, 42 genes directly interacted with each other, with *LCK* and *STAT3* having the highest degree of connectivity (Fig. 5b). Among the functional partners of the DEGs, the Ubiquitin C (*UBC*) gene was most highly connected in the network by interacting with 112 other genes, 110 of which were DEGs (Fig. 5a).

### Validation of the meta-analysis

To evaluate the reproducibility of our results, we validated the 407 DEGs with log2 fold changes greater than 1.5 in four additional microarray datasets (GSE42834, GSE56153, GSE31348 and GSE36238). The 407 genes yielded a clear separation of PTB and control for all four datasets in PCA (Additional file 7a). Using PLS-DA, the 407 genes showed good sensitivity (above 83%) and specificity (above 82%) in all four independent datasets, and had a significantly greater AUC than a random set of 407 genes in all datasets except GSE36238, which had a small sample size (Additional file 7b). Accordingly, R^2^ and Q^2^ measures of model quality and predictability, respectively, were markedly greater for the 407 gene set than the random gene set in all four datasets (Additional file 7c).

We also performed a separate meta-analysis on the in vitro datasets available for *Mtb* infection. In total, 2535 and 2911 DEGs were identified for dendritic and THP-1 cells datasets respectively, from which 129 and 192 pathways were significantly enriched (FDR-adjusted *P* < 0.01, Additional file 8). Of them, 335 genes (13.2%) in dendritic datasets and 414 (14.2%) genes in THP-1 datasets overlap with the 1655 DEGs in patient blood expression signature (Additional files 8, 9). Some 28 pathways (15.6%) in dendritic datasets and 44 pathways (22.9%) in THP-1 datasets were shared with patient blood datasets, including key pathways of *IFN* type I, *PKR* and *PD-1* signaling (Table 2, Additional file 9). We noted that DEGs shared with patient blood profiles had a significantly higher magnitude of fold change both in dendritic and THP-1 cells than the rest of DEGs (dendritic: average absolute fold change 1.903 versus 1.639, t-test *P* = 8.2e-5, THP-1: 0.802 versus 0.735, t-test *P* = 9.2e-5). MetaCore pathway enrichment of the 335 genes shared between blood and dendritic cells identified several dendritic cell related pathways such as ‘Antigen presentation by MHC class I’ and ‘Role of *HMGB1* in dendritic cell maturation and migration’ among the most significant pathways (Additional file 8).

To explore possible prognostic value of the DEGs, we performed a Kaplan-Meier survival analysis using the top 50 significant DEGs in the meta-analysis on the largest lung cancer cohort in SurvExpress [23], because significant comorbidities have been observed between lung cancer and TB [24]. The top 50 genes showed a significant prognostic feature on lung cancer survival with a Risk Group Hazard Ratio 2.01 (*P* = 4.16e-13, Additional file 10).

### Targets with human genetic evidence

Early selection of drug targets with human genetic support for involvement with disease pathology could significantly increase the success rate of late phase clinical development [34]. We sought genetic evidence for the 407 DEGs in public genome-wide association study (GWAS) datasets by looking at their individual chromosomal proximity to TB-associated SNPs. A total of 547 TB-related SNPs (genome wide significant *P* < 5e-8) were identified in GRASP, a deeply extracted and annotated GWAS database [27]. For each SNP, we overlapped its LD region with the genomic locations of all 407 DEGs. We also searched for regulatory variants for the genes using their enhancer regions from FANTOM5 [28]. Any overlap between the region in LD with a SNP and the locations of a DEG or its FANTOM5 enhancers would suggest a potential association between the SNP and that gene. Of the 407 DEGs, 48 genes were proximal to at least one TB-related SNP (Table 3). The most significant variant was rs3948464 (*P* = 5.9e-37), located within the gene *SP110* on chromosome 2, which is a known host genetic susceptibility for TB [35]. This was followed by rs3129750 (*P* = 2.5e-22) proximal to *HLA*-*DPA1*, *PSMB8*, *PSMB9*, *TAP1* and *TAP2* genes located on chromosome 6.

**Table 3:** List of 48 DEGs in the meta-analysis proximal to TB-associated SNPs

DEG	Fold change in meta-analysis	FDR *P*-value in meta-analysis	Most significant SNP	SNP *P*-value
*SP110*	1.545	8.02e-04	rs3948464	5.90e-37
*HLA-DPA1*	1.566	7.24e-03	rs3129750	2.51e-22
*PSMB8*	1.890	5.14e-08	rs3129750	2.51e-22
*PSMB9*	2.386	3.94e-06	rs3129750	2.51e-22
*TAP1*	2.332	1.03e-06	rs3129750	2.51e-22
*TAP2*	1.918	3.54e-04	rs3129750	2.51e-22
*GBP5*	3.432	1.33e-08	rs2146340	6.90e-19
*CABIN1*	−1.740	3.62e-03	rs2154594	7.31e-19
*WDR6*	−1.606	4.21e-06	rs1134591	1.62e-15
*SNX10*	2.000	3.23e-06	rs3814095	7.76e-15
*CD5*	−1.611	0.00e + 00	rs10897125	1.01e-14
*CD6*	−1.940	4.95e-03	rs10897125	1.01e-14
*TIMM10*	1.971	2.16e-08	rs2649662	2.11e-14
*WARS*	2.345	1.99e-07	rs1009812	2.69e-14
*PSMB10*	1.793	3.97e-05	rs12102971	3.17e-14
*MS4A4A*	1.882	3.51e-06	rs10750936	3.92e-14
*C2*	1.823	4.89e-12	rs2532929	4.87e-14
*MICB*	1.580	4.85e-04	rs2532929	4.87e-14
*COX19*	−1.504	1.46e-03	rs11761941	4.64e-12
*FBXO31*	−1.698	3.59e-06	rs10779243	8.09e-12
*LAP3*	2.956	1.33e-08	rs10939733	2.11e-11
*CIRBP*	−1.523	6.03e-04	rs2285899	2.41e-11
*PCSK7*	−1.824	8.45e-04	rs1060211	3.98e-11
*BLK*	−1.715	7.73e-09	rs2254546	8.30e-11
*GBP2*	2.435	1.48e-05	rs12121223	3.38e-10
*GBP3*	1.561	1.46e-04	rs12121223	3.38e-10
*OAS1*	1.832	3.46e-05	rs10774671	3.72e-10
*BAZ1A*	1.540	3.94e-03	rs799486	4.69e-10
*ZBTB40*	−1.500	6.38e-03	rs7544210	5.12e-10
*CSTA*	1.771	1.14e-04	rs10934559	5.86e-10
*PBX4*	−1.880	6.00e-06	rs1859287	7.42e-10
*CCR7*	−2.382	5.14e-08	rs11659024	7.69e-10
*C6orf136*	−1.502	1.34e-03	rs1317834	1.13e-09
*MDC1*	−1.595	2.24e-09	rs1317834	1.13e-09
*ITGAM*	1.607	5.77e-07	rs749671	1.17e-09
*CD19*	−1.658	1.60e-03	rs7185232	1.21e-09
*ACSS1*	−1.717	7.23e-06	rs6138553	1.79e-09
*SQRDL*	2.003	1.62e-09	rs1980288	2.82e-09
*SCO2*	1.909	2.96e-03	rs12148	2.92e-09
*ACTA2*	1.553	8.68e-04	rs1800682	3.81e-09
*FAS*	1.867	7.49e-06	rs1800682	3.81e-09
*GMFG*	1.836	2.11e-03	rs10412931	6.74e-09
*MAP4K1*	−1.981	4.81e-03	rs10775533	8.63e-09
*ENTPD1*	1.871	2.26e-05	rs10882657	9.31e-09
*KIF1B*	1.506	2.03e-04	rs11121555	2.21e-08
*PGD*	1.768	1.49e-05	rs11121555	2.21e-08
*EEF1D*	−1.584	2.57e-03	rs11136344	2.56e-08
*OLIG1*	−1.528	1.13e-05	rs1044213	3.89e-08

The DEGs were ranked by the *P*-value of the most significant SNPs

### Drug repurposing opportunities

We queried each of the 407 DEGs against the DrugBank database [30] in order to identify any launched drugs targeting these genes that might be potentially repurposed against TB. A total of 19 drug-target links were identified between 14 public drugs and 16 DEGs (Table 4). Each compound was associated with one gene target except for Carfilzomib, which is an inhibitor for multiple proteosome components (*PSMB2*, *PSMB8*, *PSMB9* and *PSMB10*), and Intravenous Immunoglobulin (IVIg), which targets three DEGs (*C5*, *FCGR2A* and *FCGR3A*) as either a receptor binder or antagonist.

**Table 4:** List of launched drugs in DrugBank targeting DEGs in the meta-analysis

DEG	Fold change	Drug name	Pharmacological action	Indication
*PSMB8*	1.890	Carfilzomib	Inhibitor	Multiple myeloma
*PSMB9*	2.386	Carfilzomib	Inhibitor	Multiple myeloma
*PSMB10*	1.793	Carfilzomib	Inhibitor	Multiple myeloma
*PSMB2*	1.590	Carfilzomib	Inhibitor	Multiple myeloma
*FCGR2A*	1.517	Intravenous Immunoglobulin	Antagonist	Immunodeficiencies, autoimmune and inflammatory disorders
*FCGR3A*	1.632	Intravenous Immunoglobulin	Antagonist	Immunodeficiencies, autoimmune and inflammatory disorders
*C5*	1.981	Intravenous Immunoglobulin	Binder	Immunodeficiencies, autoimmune and inflammatory disorders
*C5*	1.981	Eculizumab	Antibody	Antibody against C5
*IL1B*	1.629	Canakinumab	Binder	Familial Cold Autoinflammatory Syndrome and Muckle-Wells Syndrome
*IL1B*	1.629	Gallium nitrate	Antagonist	Hypercalcemia, non-hodgkin’s lymphoma
*JAK2*	2.756	Ruxolitinib	Inhibitor	High-risk myelofibrosis
*JAK2*	2.756	Tofacitinib	Antagonist	Rheumatoid arthritis
*TLR7*	1.899	Hydroxychloroquine	Antagonist	Malaria
*CD19*	−1.658	Blinatumomab	Activator	Refractory B-cell precursor acute lymphoblastic leukemia
*CD247*	−1.586	Muromonab	Binder	Prevention of organ rejection
*CD274*	1.674	Atezolizumab	Antibody	Urothelial carcinoma
*IL23A*	−1.621	Ustekinumab	Antibody	Management of moderate to severe plaque psoriasis
*POLB*	1.888	Cytarabine	Inhibitor	Acute non-lymphocytic leukemia
*S1PR1*	−1.956	Asfotase Alfa	Agonist	Hypophosphatasia

CMAP is another computational tool for drug repurposing which utilizes the anti-correlation relationships between gene expression signatures in diseases and drug perturbations [31]. We used the PTB gene expression signature to recover the anti-correlation signatures of ~5000 small-molecule compounds and ~3000 genetic reagents from the Broad Institute CMAP library. Thirteen compounds were found to have a significantly anti-correlated signature to the PTB signature (FDR-adjusted *P* < 0.05, Specificity < 0.1, Table 5). Six of these drugs were cardiovascular related therapeutics, including two sodium channel blockers (Disopyramide, Mephenytoin), two voltage-gated calcium channel blockers (Flunarizine, Adenosine Phosphate) and one ATP-sensitive potassium channel blocker (Acetohexamide) (Table 5).

**Table 5:** List of significant public compounds in CMAP analysis

Compound	*P*-value	Specificity	Therapy area	Pharmacological action	Indication	Target
Disopyramide	0	0.0006	Cardiovascular	Sodium channel blocker	Arrhythmia	*SCN5A, ORM1*
Biperiden	0.0005	0.0984	Neurological	Muscarinic acetylcholine receptor antagonist	Parkinsonism	*CHRNA2, CHRM1*
Remoxipride	0.0036	0.0164	Neurological	Dopamine receptor D2 antagonist	Schizophrenia	*DRD2*
Suramin sodium	0.0101	0.0129	Anti-infective	Topoisomerase inhibitor	African trypanosomiasis	*P2RY2, SIRT5, FSHR*
Flunarizine	0.0133	0.0119	Cardiovascular	Voltage-gated calcium channel blocker; sodium channel antagonist	Migraine, epilepsy	*HRH1, CACNA1G, CACNA1H, CACNA1I, CALM1*
Adenosine phosphate	0.019	0.0007	Cardiovascular	Calcium channel blocker	Arrhythmia	Unknown
Ranitidine	0.0332	0.049	Miscellaneous	Histamine receptor H2 antagonist	Peptic Ulcer	*HRH2*
Chloropyramine	0.0355	0.0523	Miscellaneous	Histamine H1 receptor antagonist	Antiallergic agent	*HRH1*
Acetohexamide	0.0408	0.0508	Cardiovascular	Blocking of ATP-sensitive K+ channel	Diabetes mellitus type 2	*KCNJ1*
Dobutamine	0.0411	0.0665	Cardiovascular	Adrenoreceptor agonist (beta1)	Cardiac decompensation	*ADRB1*
Mephenytoin	0.0441	0.0444	Cardiovascular	Sodium channel inhibitor	Seizures	*SCN5A, ALB*
Testosterone	0.0476	0.0783	Miscellaneous	Androgen receptor agonist	Hypogonadism	*AR, ALB, SHBG, NPPB*
Dienestrol	0.0483	0.0982	Miscellaneous	Estrogen	Atrophic vaginitis	*ESR1*

## Discussion

We performed a meta-analysis on public human microarray datasets to identify host genes and pathways important for TB that might be amenable to therapeutic modulation. In our study, a plethora of DEGs and pathways were identified in PTB infection. In comparison, no known host process was identified in LTB. This is consistent with a paucity of host immune response to *Mtb* during the latent stage [4] and highlights the challenges of LTB detection and treatment.

Blood gene expression represents a mixture of immune cell subpopulations, which renders the question whether expression signatures derived from whole blood or PBMCs could recapitulate those of individual cell types. Overall, the similarity between gene expression signatures of *Mtb* challenged patient blood and the immune cell subsets was modest, which is in agreement with previous observations of large variation in expression profiles among different immune cell types [36, 37]. Nevertheless, dendritic or THP-1 cell-types DEGs shared with those found in blood have significantly higher fold changes compared to non-shared DEGs, which implicates that genes bearing a stronger expression signal in individual cell type could be more readily detected in the broader blood expression profiles. Moreover, the finding that common genes between blood and dendritic cells were enriched in cell type specific pathways indicates that blood gene expression signatures at least partially recapitulate the major signals from these immune cell types when subjected to *Mtb* exposure.

Our meta-analysis supports multiple known host responses to *Mtb* infection and suggests several novel host targets and drug repurposing opportunities. We identified numerous *IFN*-inducible genes with the *IFN*-signaling pathway most significantly enriched, which is consistent with previous findings on the predominant response of this pathway to TB infection [9, 10]. Among the *IFN*-inducible genes, *HLA-DPA1*, *PSMB8*, *PSMB9*, *TAP1* and *TAP2* were proximal to rs3129750, a highly significant genetic variant associated with TB (*P* = 2.51e-22), providing genetic support for these genes as potential host targets. *PSMB8* and *PSMB9* are the targets of Carfilzomib, a proteasome inhibitor for multiple myeloma.

We found evidence for TB activation of several pathways more commonly associated with other non-infectious diseases, which could be informative for drug repurposing opportunities. For example, the *LRRK2* pathway linked to Parkinson’s disease and *PD-1* signaling pathway in immuno-oncology were both significantly enriched during PTB infection. *LRRK2* was a highly significant DEG (fold change = 1.73, FDR-adjusted *P =* 1.7e-4) in the meta-analysis and directly interacts with seven other DEGs in the pathway, including two components in the NRON complex through which *LRRK2* inhibits the immune response transcription factor *NFAT1* [38]. The *LRRK2* gene is associated with both Parkinson’s disease and susceptibility to *Mycobacterium leprae* infection [39, 40]. A recent patient cohort analysis revealed a 1.38-fold risk of Parkinson’s disease in TB patients independent of other clinical factors, suggesting the co-morbidity between the two diseases [41]. In addition, 58 genetic variants proximal to the 407 DEGs were found to be associated with Parkinson’s disease in public GWAS datasets (*P* < 1e-4, Additional file 11), thus providing some preliminary genetic evidence for potential common molecular mechanisms shared by both diseases.

*PD-1* is the key immune checkpoint receptor that mediates T-cell immuno-suppression in cancer. The *PD-1*/*PD-L*s pathway is known to inhibit T-cell effector function during PTB infection [42, 43]. Both *PD-L1* and *PD-L2* genes were significantly up-regulated, and nine DEGs in T cells were down-regulated, indicating a PD-Ls mediated immune suppression is likely active in tuberculosis. Among the existing drugs, both Muromonab and Atezolizumab target DEGs (*CD247* and *CD274*/*PD-L1*) in the pathway (Table 4). New drugs that block *PD-1*/PD-Ls interaction in order to enhance the T-cell adaptive immune response against tumors are revolutionizing oncology medicine [44]. Several anti-*CTLA-4*/*PD-1*/*TIM3*/*LAG3* compounds are under investigation as repurposing candidates against TB [45] (Table 6). Our results support the hypothesis that overcoming T-cell exhaustion could be a common therapeutic strategy for both cancer immuno-therapy and TB [46].

**Table 6:** List of TB host targets or compounds proposed in this study

Examples of current therapies under investigation			Targets and compounds proposed in this study		
Compounds	Targets/Pathways	Evidence [45]	Compounds	Targets/Pathways	Evidence
Aspirin	Arachidonic acid metabolism	Upregulation of lipoxin X4 production to reduce TNF-α levels and achieve eicosanoid balance during chronic inflammation.	*LRRK2* inhibitor	*LRRK2* pathway	*LRRK2* pathway significantly upregulated in TB. *LRRK2* genetically associated with susceptibility of *M. leprae* infection. Cormobidities between TB and Parkinson’s disease.
Anti-*CTLA4*/*PD-1*/*TIM3*/*LAG3*	Modulation of aberrant T-cell activity	Blockade of immune checkpoint pathways to restore T- and B-cell activity.	*PD-L1* inhibitor (Atezolizumab)	*PD-1*/*PD-L1* pathway	*PD-1*/*PD-L1* significantly upregulated in TB, and inhibit TB-specific T-cell and macrophage functions.
Valproic acid	Histone acetylation	Removal of acetyl groups of lysine residues on histones to allow DNA unwinding and gene transcription.	Carfizomib	*PSMB8*, *PSMB9*, *PSMB10*, *PSMB2*	*PSMB8*, *PSMB9* significantly upregulated in TB, with strong genetic association with TB infection.
Statins	Disruption of cholesterol homeostasis	Abrogates production of endogenous cholesterol.	Intraveneous Immunoglobulin (IVIg)	*FCGR2A*, *FCGR3A*, *C5*	*FCGR2A*, *FCGR3A*, *C5* significantly upregulated in TB. Efficacy of IVIg in reducing bacterial load in TB infection.
Verapamil/Carbamazepine	Modulation of ion efflux channels	Modulation of activity of voltage-gated channels to maintain cellular ionic balance and homeostasis.	Disopyramide	*SCN5A*, *ORM1*	Top compound in CMAP analysis. *SCN5A* regulates spatial and temporal calcium signaling during *Mtb* phagocytosis.
Metformin	Mitochondrial respiration	Interrupts the mitochondrial respiratory chain and induces ROS production.	Flunarizine	*HRH1*, *CACNA1G*, *CACNA1H*, *CACNA1I*, *CALM1*	Top compound in CMAP analysis. Potential efficacy in restricting *Mtb* growth.

List of TB host targets and compounds proposed in this study as well as examples of current repurposed drugs under investigation for TB host-directed therapies (full list refer to [45])

To gain insight into the functional relationships between the TB-related genes, we depict a protein-protein interaction network for the 1655 DEGs. The network was predominated by a few hub nodes that were highly connected to multiple other genes. Of them, the *UBC* gene has the highest degree of connectivity by associating with 112 genes, suggesting that *UBC*, although its expression was not significantly altered, could play a central role in TB through regulating the expression of numerous TB-related host factors. *UBC* is a stress inducible gene and is one of the four genes encoding for human ubiquitin [47]. *Mtb* is known to suppress host innate immunity through the ubiquitin system [48]. Modulating host ubiquitin pathways could be another important strategy to reactivate host innate response against *Mtb*.

Our searches of DrugBank using the 407 DEGs revealed 19 highly supported drug-target links encompassing 14 drugs and 16 targets. Among these, the drug intravenous immunoglobulin or IVIg was found targeting two Fc receptors and one complement component as either an antagonist or a receptor binder, thus supporting the phagocytic pathway of *Mtb* as a potential druggable target. IVIg is used to treat patients with primary immunodeficiency and has been applied to a wide range of autoimmune and inflammatory conditions [49]. An in vivo mouse experiment showed the efficacy of high-dose IVIg in substantially reducing the bacterial load during *Mtb* infection [50]. Our results suggest that this could be accomplished through enhancing the complement system and inhibiting Fc receptors which subsequently initiate phagocytosis to clear the bacillus [51].

From our CMAP analysis, we identified several public compounds that could be potentially repurposed for TB. Of particular interest are two sodium channel blockers and two voltage-gated calcium channel blockers. It has been suggested that voltage-gated calcium channels regulate the host immune response to *Mtb* by enhancing the pro-inflammatory gene expression and activating T-cells [52]. The sodium channel NaV1.5 encoded by *SCN5A*, the drug target of Disopyramide that was top ranked in our analysis, regulates the spatial and temporal calcium signaling during mycobacterial phagocytosis [53]. Indeed, studies screening host-targeted inhibitors demonstrated the efficacy of several calcium and sodium channel blockers in restricting mycobacterial growth both in vitro and in macrophages [54]. Sodium or calcium channel blockers such as Carbamazepine and Verapamil are under investigation for TB treatment [45] (Table 6). Further experimental validation of both IVIg and ion channel blockers is merited to explore their application in TB host-directed therapies.

Previously, we performed meta-analysis of human gene expression datasets to identify host targets and drug repositioning opportunities against respiratory bacterial and viral infections [13, 14]. In this study, we applied an overall similar strategy to identify same opportunities for the treatment of TB. Despite an overall common data analysis methodology, there are multiple novelties in the current study compared to our early work in respiratory bacterial and viral infections. First, instead of using a vote-counting method with arbitrary cutoffs on the number of studies in which a gene was declared significant, we employed the combining effect size method of the statistical meta-analysis which essentially yields one single biologically interpretable measure - the pooled effect size (and *P*-value) of differential expression. The combining effect size method has been suggested as the most comprehensive approach for meta-analysis of two-class gene expression microarrays [55]. Second, we assessed the robustness of the meta-analysis results by cross-validation in other independent patient blood datasets, and comparison to in vitro cell type specific datasets. Third, additional genetic evidence from public GWAS datasets was used in this study to further prioritize individual DEGs as potential therapeutic targets for TB. Fourth, a PPI network was employed to obtain protein-protein interaction modules important in TB and generate novel targets (i.e. *UBC*) that may play a role in regulating the expression of multiple TB-related host factors. Fifth, with respect to drug repurposing analysis, CMAP analysis was performed to generate additional hypotheses based on anti-correlation relationships between gene expression signatures in diseases and drug perturbations (Table 5).

## Conclusions

In summary, our meta-analysis provides new insights into host genes and pathways important for TB infection and brings forth potential drug repurposing opportunities for host-directed therapies (perhaps as potential combination therapies with anti-mycobacterium drugs). The potential host targets and compounds proposed in the study were listed in Table 6. Several testable hypotheses from our study are: 1) *LRRK2* inhibition reduces *Mtb* load in macrophages; 2) blocking *PD-L1* reactivates T-cell exhaustion against *Mtb* infected dendritic cells and; 3) ion-channel blockers as repurposed drugs to enhance T-cell activation and suppress *Mtb* growth in macrophages. Experimental validation of these hypotheses would be the next step taking forward the proposed targets and compounds into the drug development pipeline.

## Additional files

Additional file 1: The quality control analysis for (A) GSE56153 and (B) GSE19435. The kernel density plot and heatmap for within-group pairwise correlation are shown for both datasets. Both a noisy kernel density plot and extremely low within-group pairwise correlation were observed for GSE56153. (PDF 827 kb)

Additional file 2: An example of quality control analysis for (A) GSE19439 and (B) GSE34608. Within group pairwise correlation and MAD score plots are shown for both datasets. Outlier samples are highlighted in red and indicated by arrows. (PDF 455 kb)

Additional file 3: List of the 85 GEO datasets available for TB-related host responses at the time of the study, their inclusion (color shaded) or exclusion in the meta-analysis and the reasons for their exclusion. (XLSX 23 kb)

Additional file 4: PCA plots before (A) and after (B) batch effect correction. (PDF 422 kb)

Additional file 5: List of 1655 DEGs and their fold changes and FDR *P*-values in the meta-analysis. (XLSX 73 kb)

Additional file 6: Pathway map for “*IFN* type I signaling pathway”. Significant up-regulation of genes was denoted as up-pointing bars colored in red, and significant down-regulation of genes was denoted as down-pointing bars colored in blue. The length of the colored bar was proportional to the fold change of the gene in the meta-analysis. (PDF 1079 kb)

Additional file 7: Validation of the 407 DEGs in four independent datasets. (A). PCA using the 407 DEGs showed a clear separation of PTB and control samples in all four datasets. PLS-DA using the 407 DEGs showed significantly better model performance in classifying PTB and control samples than a random set of 407 genes in terms of (B) Area under ROC and (C) R^2^ and Q^2^. (PDF 614 kb)

Additional file 8: List of DEGs and pathways in the meta-analysis of in vitro dendritic and THP-1 datasets, the DEGs shared with patient blood datasets, and the enriched MetaCore pathways from the shared DEGs. (XLSX 292 kb)

Additional file 9: Venn diagram for the overlap in DEGs and pathways between patient blood and in vitro dendritic and THP-1 datasets. (PDF 370 kb)

Additional file 10: Kaplan-Meier survival analysis plot showing a significant prognostic feature of the top 50 DEGs in the meta-analysis on the survival of the largest lung cancer cohort in SurvExpress [23]. (PDF 163 kb)

Additional file 11: List of 58 Parkinson’s disease-associated genetic variants proximal to the 407 DEGs. (XLSX 12 kb)

## Abbreviations

CMAP: Connectivity Map
DEG: Differentially expressed gene
FDR: False discovery rate
GEO: Gene expression omnibus
GO: Gene ontology
GRASP: Genome-wide repository of associations between SNPs and phenotypes
GWAS: Genome-wide association studies
IVIg: Intravenous Immunoglobulin
LD: Linkage disequilibrium
*LRRK2*: Leucine-rich repeat kinase 2
LTB: Latent tuberculosis
MAD: Median absolute deviation
MHC: Major histocompatibility complex
*Mtb*: *Mycobacterium tuberculosis*
PBMC: Peripheral blood mononuclear cell
PCA: Principal component analysis
*PKR*: Protein kinase R
PTB: Active pulmonary tuberculosis
TB: Tuberculosis
