# Supplementary figures and images for: Meta-analysis of human gene expression in response to Mycobacterium tuberculosis infection reveals potential therapeutic targets
Source: BMC Syst Biol. 2018 Jan 10;12:3. doi: 10.1186/s12918-017-0524-z (PMC5763539; doi:10.1186/s12918-017-0524-z)

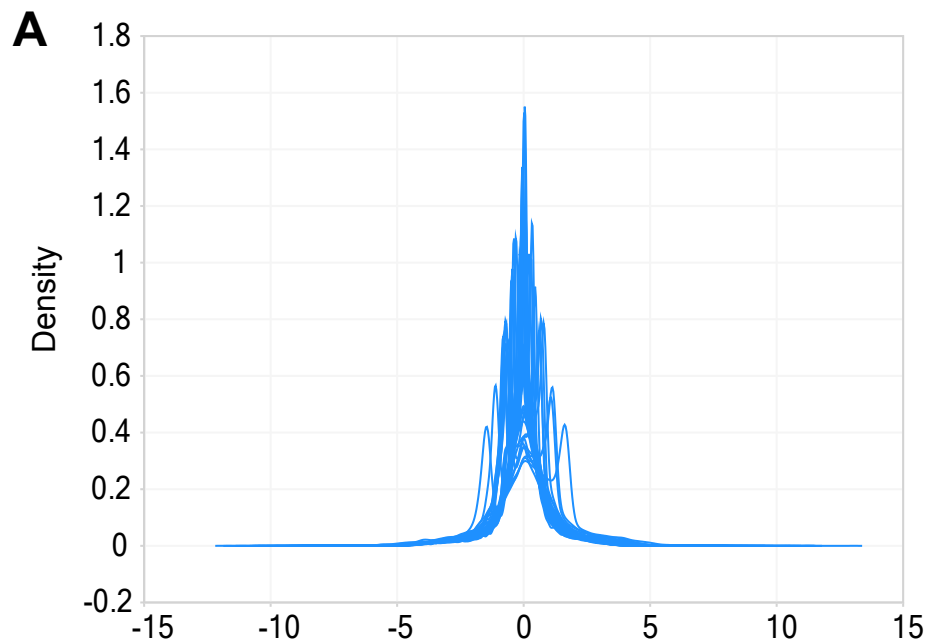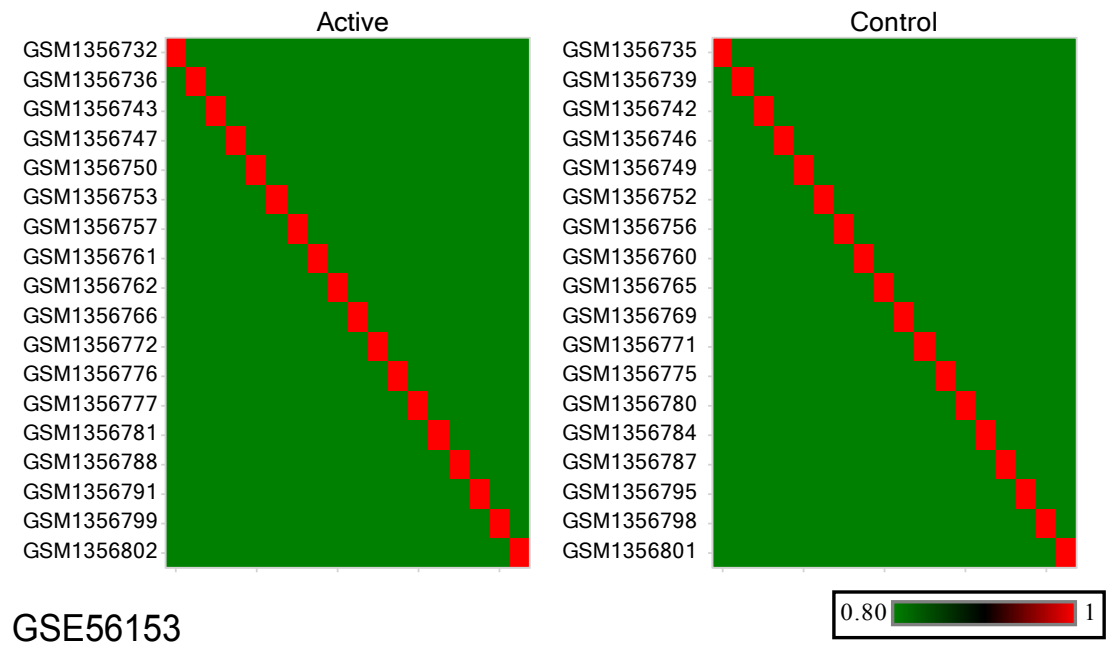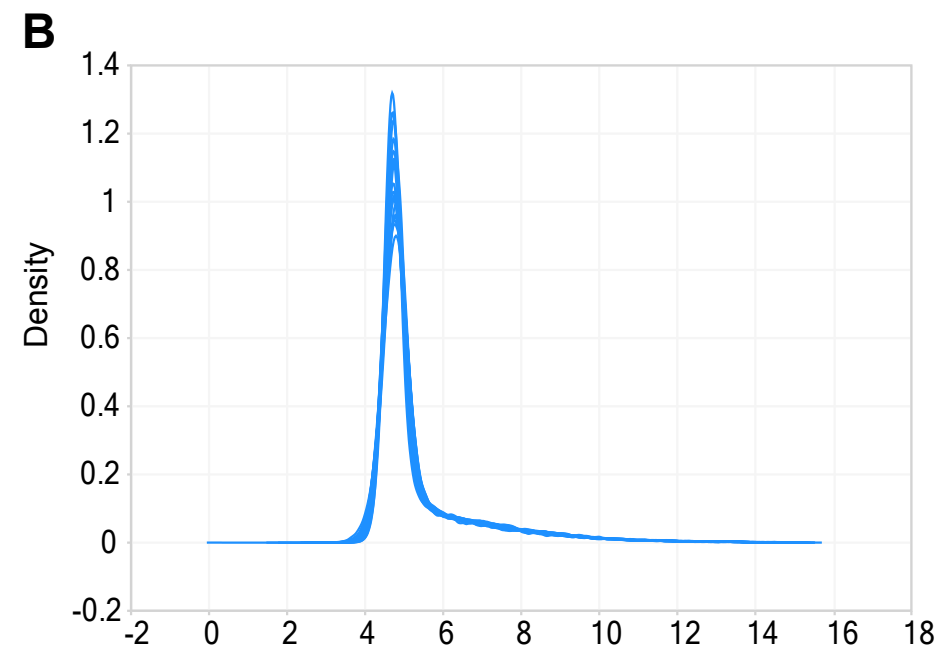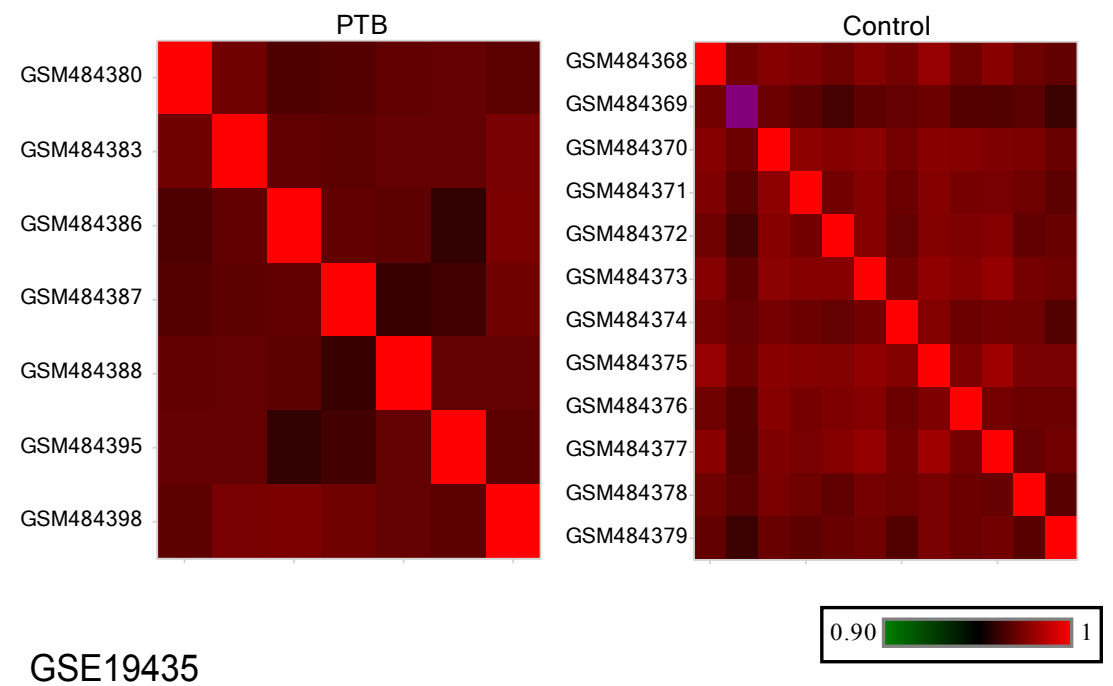

Supplement: Supplementary file 1 — The quality control analysis for (A) GSE56153 and (B) GSE19435. The kernel density plot and heatmap for within-group pairwise correlation are shown for both datasets. Both a noisy kernel density plot and extremely low within-group pairwise correlation were observed for GSE56153. (PDF 827 kb) [file 12918_2017_524_MOESM1_ESM.pdf]

**A**

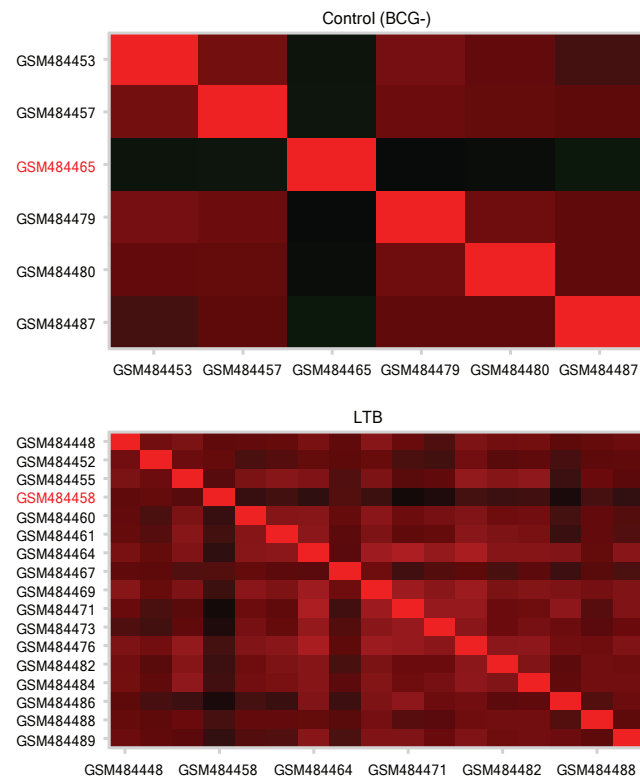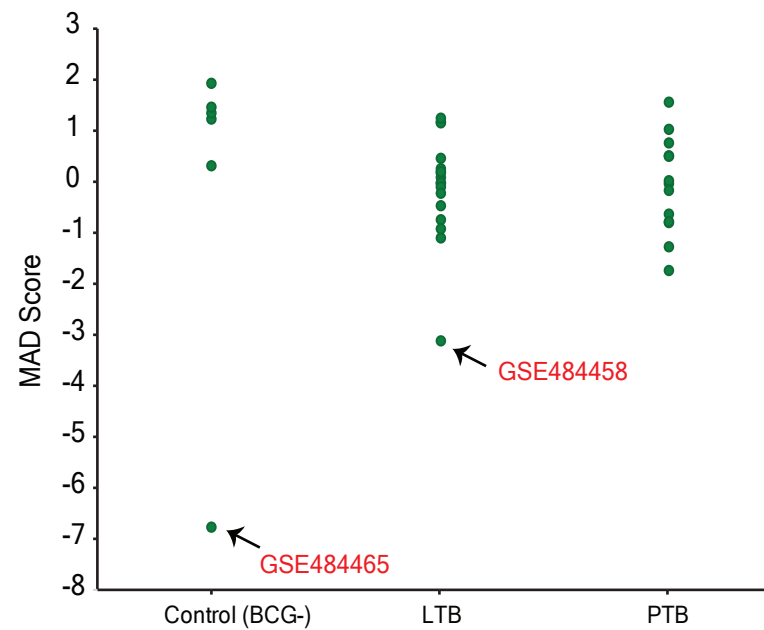

GSE19439

**B**

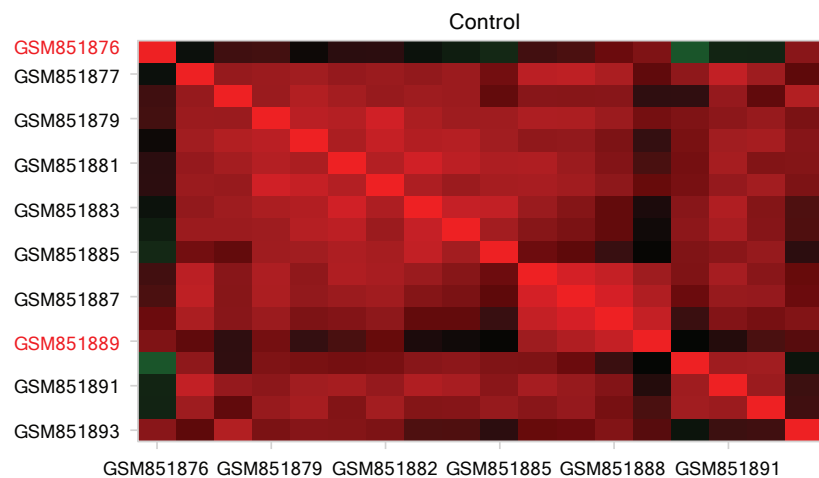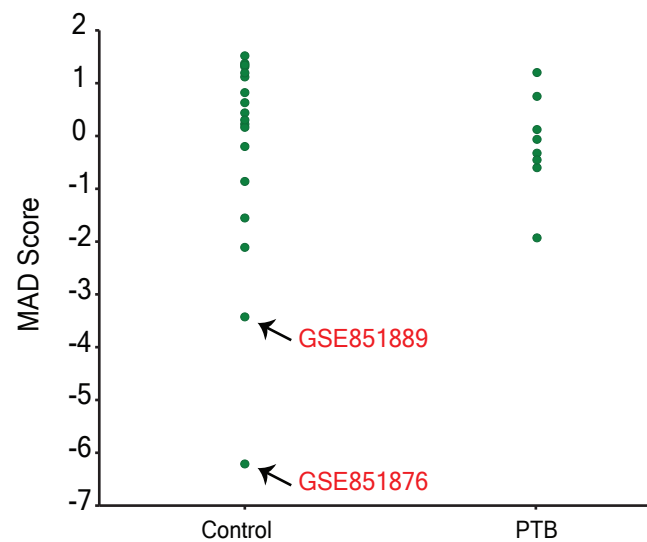

GSE34608

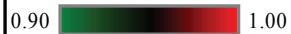

Supplement: Supplementary file 2 — An example of quality control analysis for (A) GSE19439 and (B) GSE34608. Within group pairwise correlation and MAD score plots are shown for both datasets. Outlier samples are highlighted in red and indicated by arrows. (PDF 455 kb) [file 12918_2017_524_MOESM2_ESM.pdf]

**A**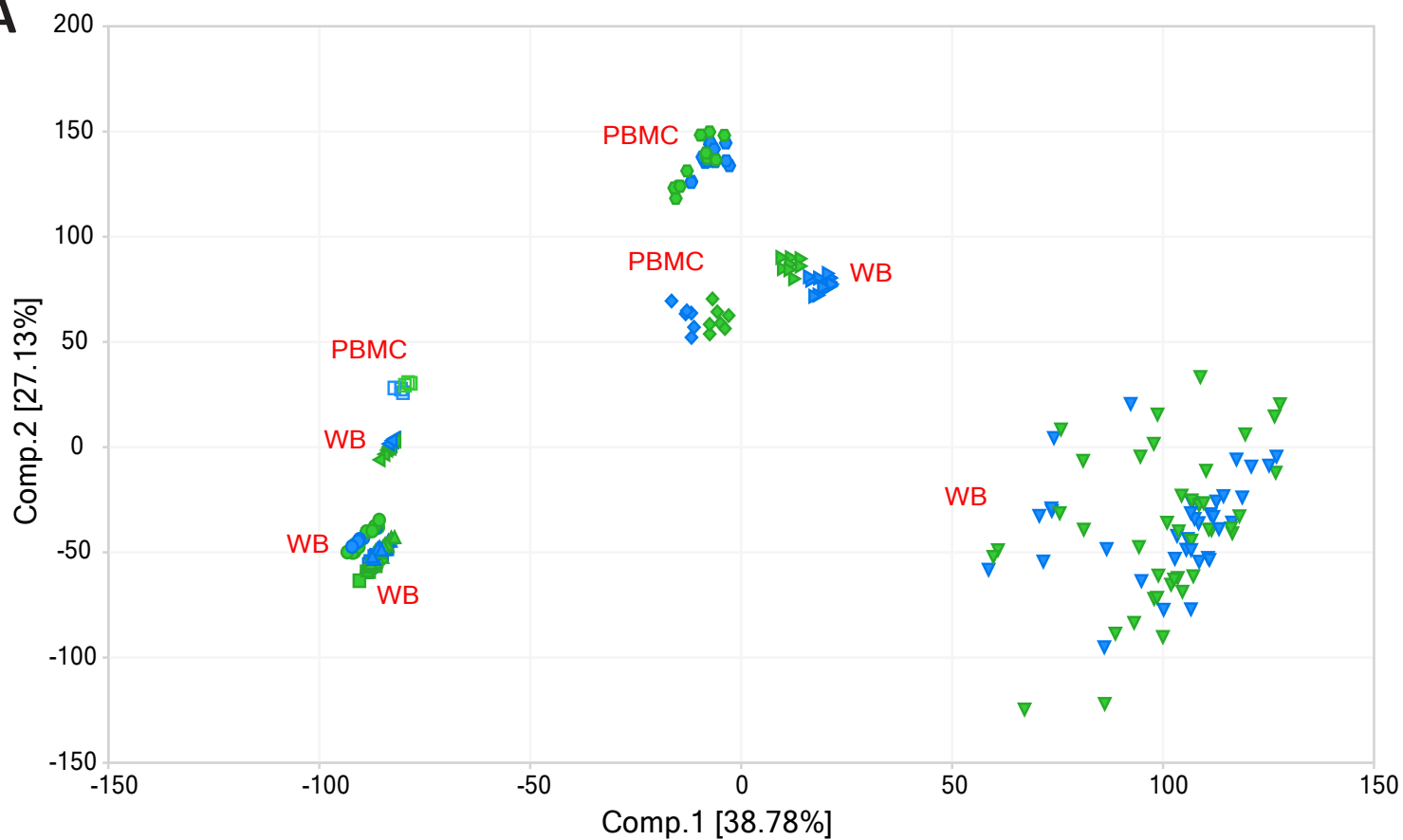**B**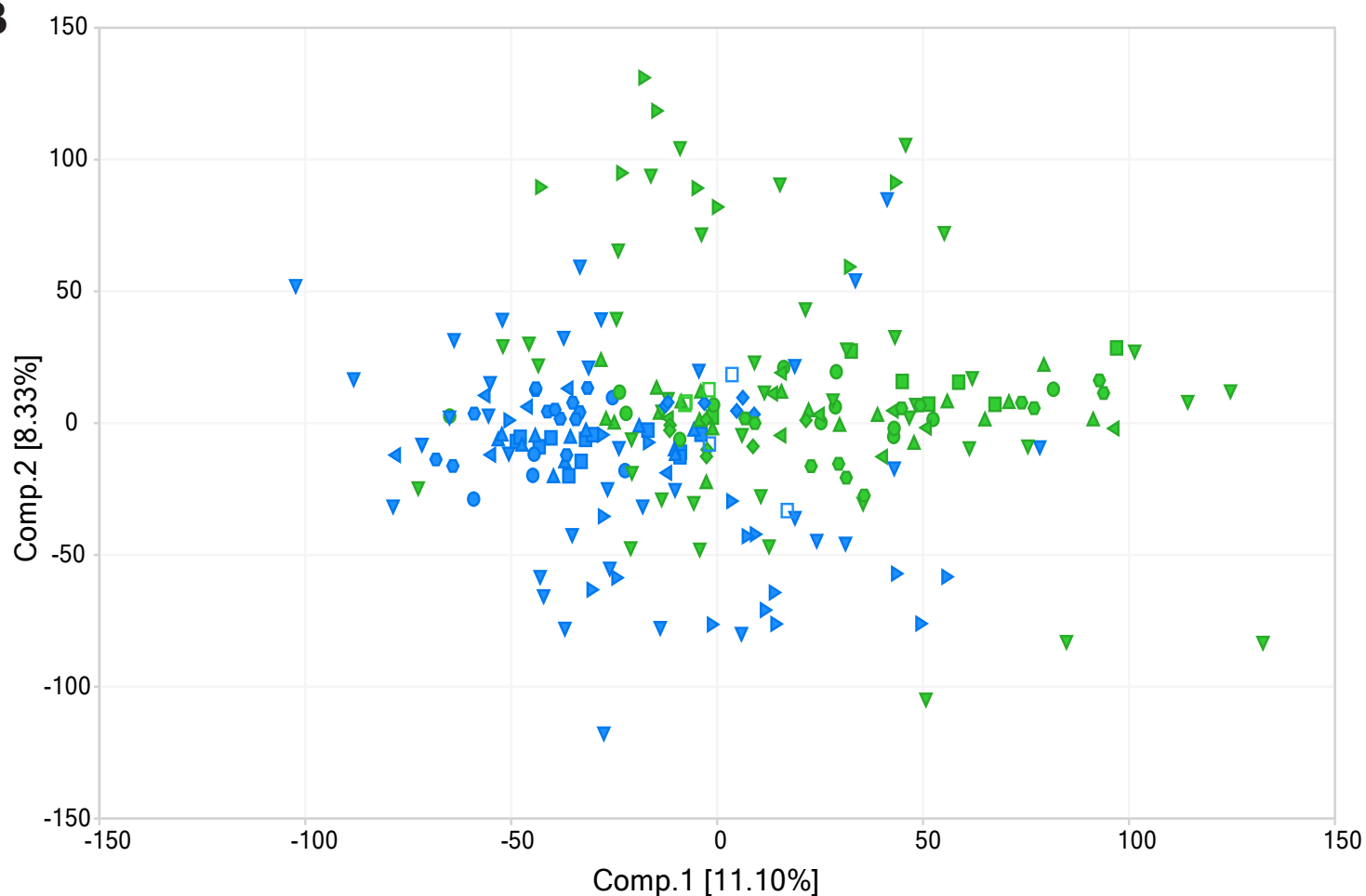

WB: Whole Blood  
PBMC: Peripheral Blood Mononuclear Cells

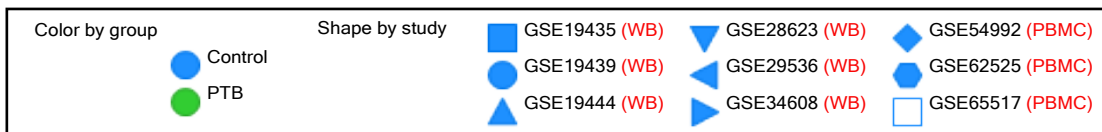

Supplement: Supplementary file 4 — PCA plots before (A) and after (B) batch effect correction. (PDF 422 kb) [file 12918_2017_524_MOESM4_ESM.pdf]

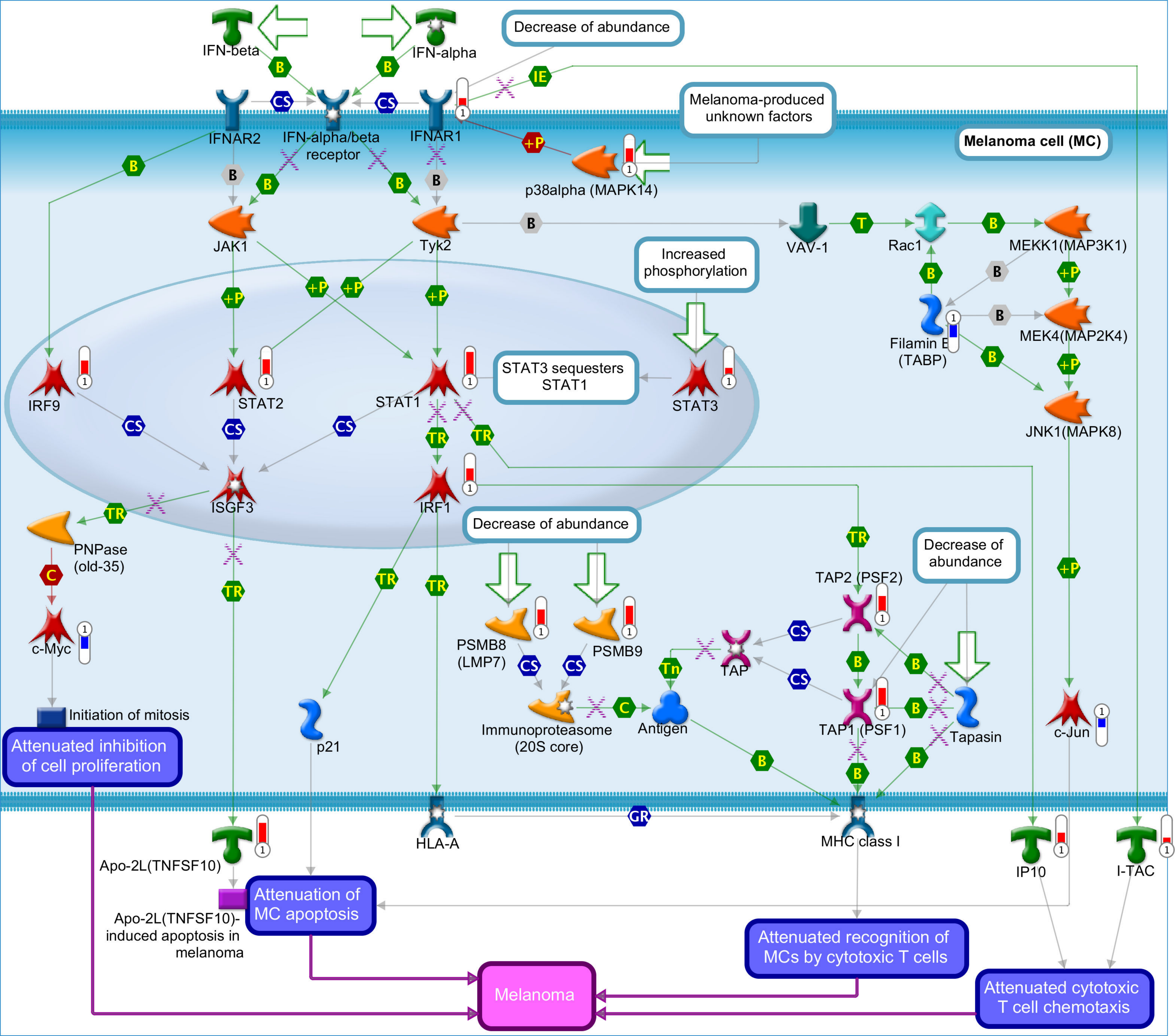

Supplement: Supplementary file 6 — Pathway map for “IFN type I signaling pathway”. Significant up-regulation of genes was denoted as up-pointing bars colored in red, and significant down-regulation of genes was denoted as down-pointing bars colored in blue. The length of the colored bar was proportional to the fold change of the gene in the meta-analysis. (PDF 1079 kb) [file 12918_2017_524_MOESM6_ESM.pdf]

**A**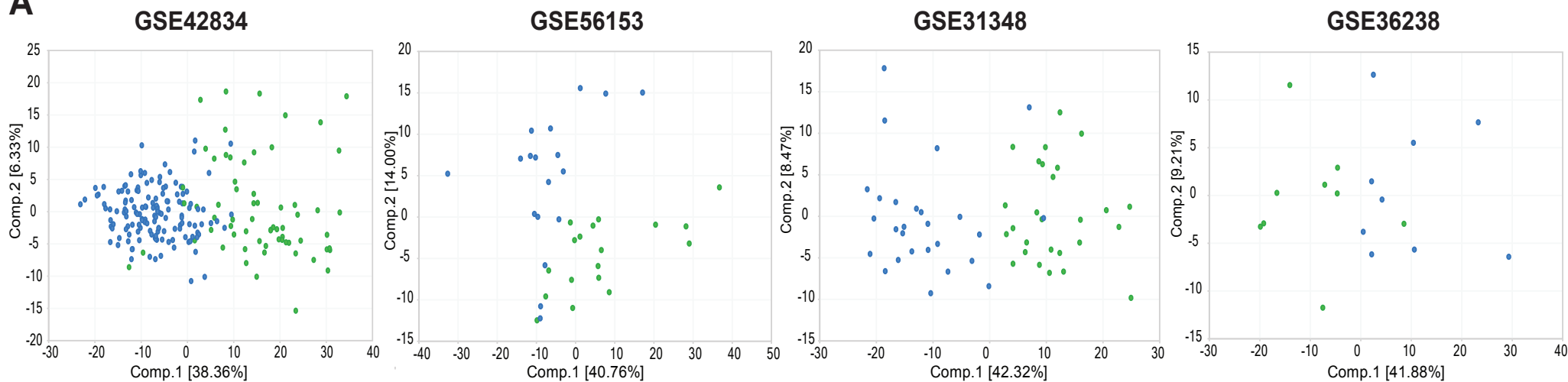**B**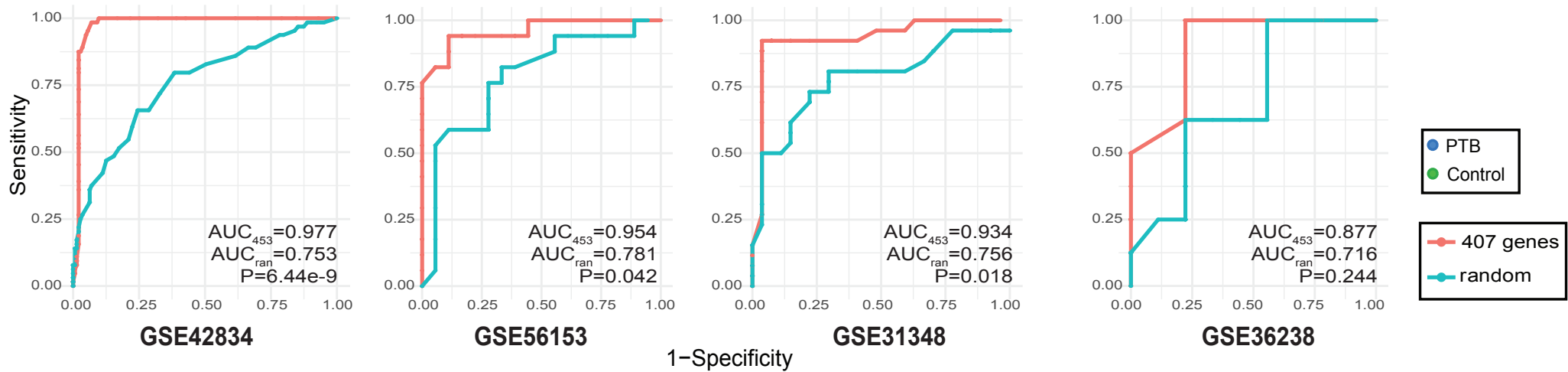**C**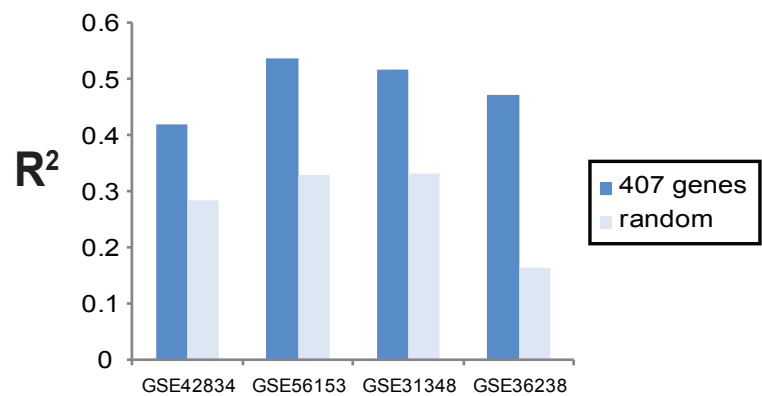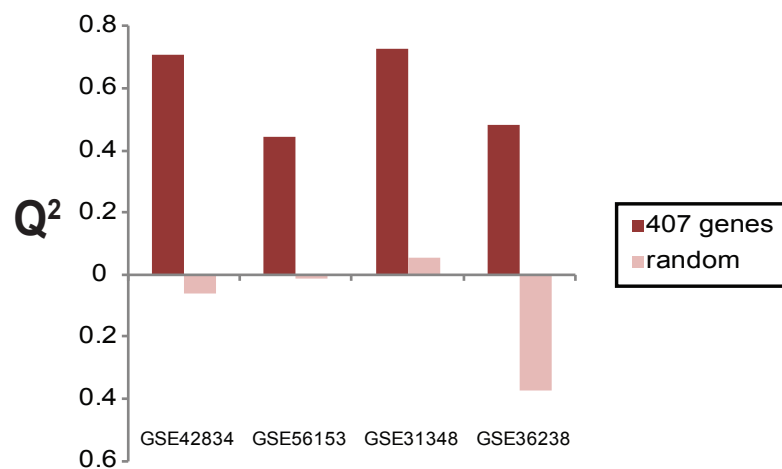

Supplement: Supplementary file 7 — Validation of the 407 DEGs in four independent datasets. (A). PCA using the 407 DEGs showed a clear separation of PTB and control samples in all four datasets. PLS-DA using the 407 DEGs showed significantly better model performance in classifying PTB and control samples than a random set of 407 genes in terms of (B) Area under ROC and (C) R2 and Q2. (PDF 614 kb) [file 12918_2017_524_MOESM7_ESM.pdf]
