# Supplementary material for: Meta-analysis of human gene expression in response to Mycobacterium tuberculosis infection reveals potential therapeutic targets
Source: BMC Syst Biol. 2018 Jan 10;12:3. doi: 10.1186/s12918-017-0524-z (PMC5763539; doi:10.1186/s12918-017-0524-z)
Supplement: Supplementary file 9 — Venn diagram for the overlap in DEGs and pathways between patient blood and in vitro dendritic and THP-1 datasets. (PDF 370 kb) [file 12918_2017_524_MOESM9_ESM.pdf]

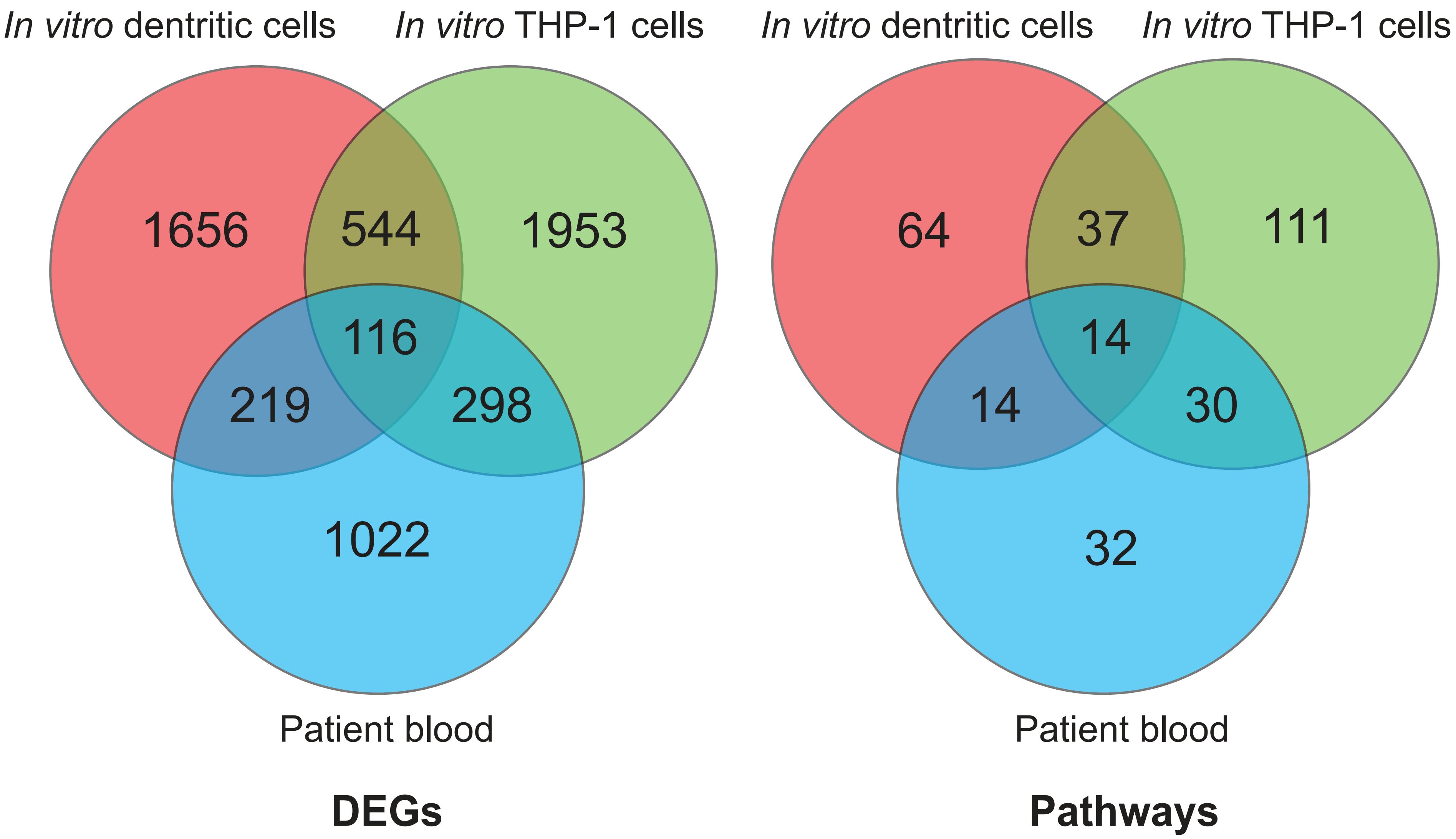
In vitro dentritic cells
In vitro THP-1 cells
1656
544
1953
116
219
298
1022
Patient blood
DEGs
In vitro dentritic cells
In vitro THP-1 cells
64
37
111
14
14
30
32
Patient blood
Pathways
