# Supplementary material for: Meta-analysis of human gene expression in response to Mycobacterium tuberculosis infection reveals potential therapeutic targets
Source: BMC Syst Biol. 2018 Jan 10;12:3. doi: 10.1186/s12918-017-0524-z (PMC5763539; doi:10.1186/s12918-017-0524-z)
Supplement: Supplementary file 10 — Kaplan-Meier survival analysis plot showing a significant prognostic feature of the top 50 DEGs in the meta-analysis on the survival of the largest lung cancer cohort in SurvExpress [23]. (PDF 163 kb) [file 12918_2017_524_MOESM10_ESM.pdf]

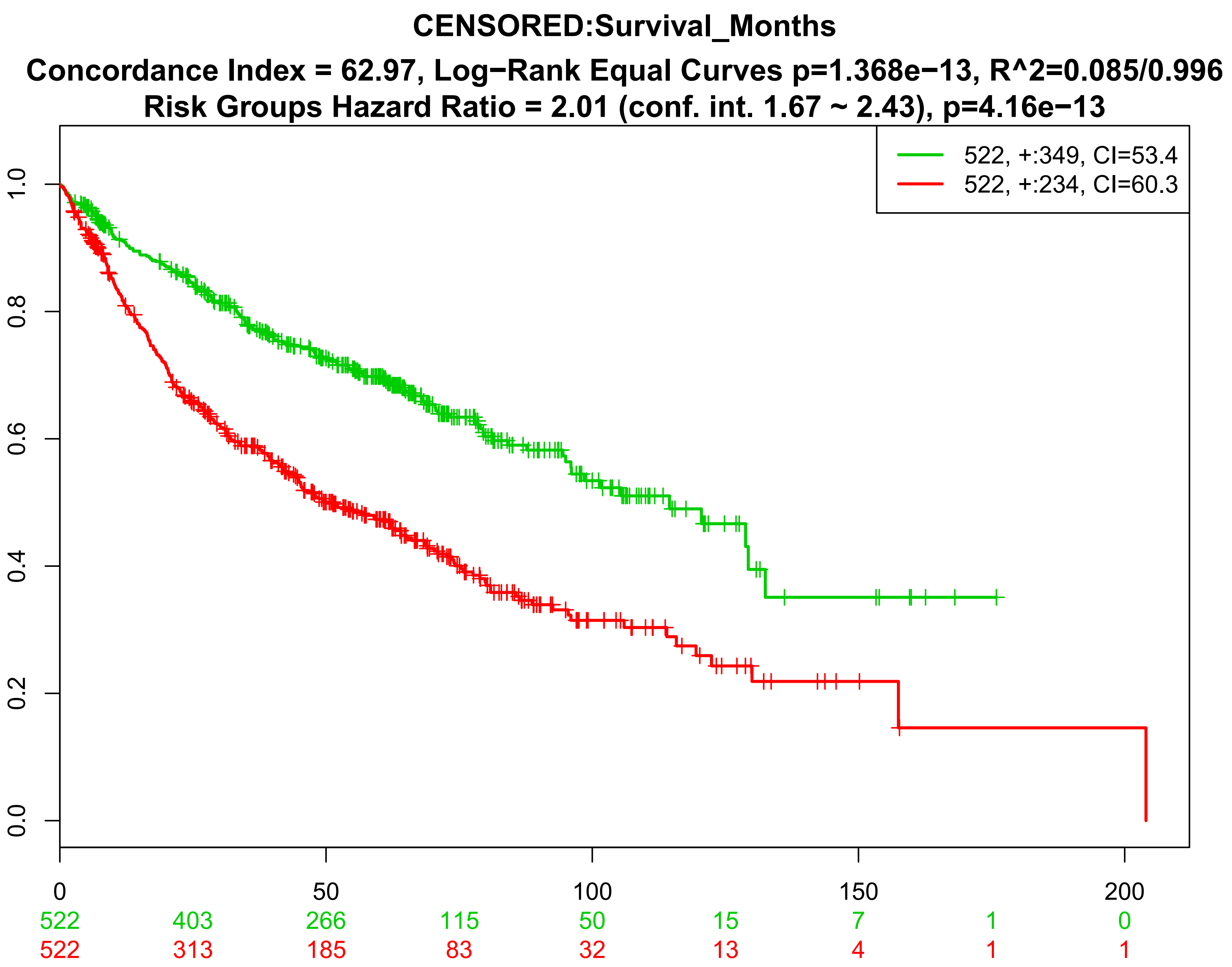
CENSORED:Survival_Months
Concordance Index = 62.97, Log−Rank Equal Curves p=1.368e−13, R^2=0.085/0.996
Risk Groups Hazard Ratio = 2.01 (conf. int. 1.67 ~ 2.43), p=4.16e−13
522, +:349, CI=53.4
522, +:234, CI=60.3
0.0
0.2
0.4
0.6
0.8
1.0
0
50
100
150
200
522 403 266 115 50 15 7 1 0
522 313 185 83 32 13 4 1 1
